# ALS-associated genes in SCA2 mouse spinal cord transcriptomes

**DOI:** 10.1093/hmg/ddaa072

**Published:** 2020-04-20

**Authors:** Daniel R Scoles, Warunee Dansithong, Lance T Pflieger, Sharan Paul, Mandi Gandelman, Karla P Figueroa, Frank Rigo, C Frank Bennett, Stefan M Pulst

**Affiliations:** 1 Department of Neurology, University of Utah, 175 North Medical Drive East, 5th Floor, Salt Lake City, UT 84132, USA; 2 Department of Biomedical Informatics, University of Utah, 421 Wakara Way, Salt Lake City, UT 84108, USA; 3 Ionis Pharmaceuticals, 2855 Gazelle Court, Carlsbad, CA 92010, USA

## Abstract

The spinocerebellar ataxia type 2 (SCA2) gene *ATXN2* has a prominent role in the pathogenesis and treatment of amyotrophic lateral sclerosis (ALS). In addition to cerebellar ataxia, motor neuron disease is often seen in SCA2, and *ATXN2* CAG repeat expansions in the long normal range increase ALS risk. Also, lowering *ATXN2* expression in TDP-43 ALS mice prolongs their survival. Here we investigated the ATXN2 relationship with motor neuron dysfunction *in vivo* by comparing spinal cord (SC) transcriptomes reported from TDP-43 and SOD1 ALS mice and ALS patients with those from SCA2 mice. SC transcriptomes were determined using an SCA2 bacterial artificial chromosome mouse model expressing polyglutamine expanded *ATXN2*. SCA2 cerebellar transcriptomes were also determined, and we also investigated the modification of gene expression following treatment of SCA2 mice with an antisense oligonucleotide (ASO) lowering *ATXN2* expression. Differentially expressed genes (DEGs) defined three interconnected pathways (innate immunity, fatty acid biosynthesis and cholesterol biosynthesis) in separate modules identified by weighted gene co-expression network analysis. Other key pathways included the complement system and lysosome/phagosome pathways. Of all DEGs in SC, 12.6% were also dysregulated in the cerebellum. Treatment of mice with an *ATXN2* ASO also modified innate immunity, the complement system and lysosome/phagosome pathways. This study provides new insights into the underlying molecular basis of SCA2 SC phenotypes and demonstrates annotated pathways shared with TDP-43 and SOD1 ALS mice and ALS patients. It also emphasizes the importance of ATXN2 in motor neuron degeneration and confirms ATXN2 as a therapeutic target.

## Introduction

Modeling of human neurological diseases in animals has revolutionized our understanding of pathogenesis and altered pathways. RNA transcriptomes from amyotrophic lateral sclerosis (ALS) models are powerful predictive tools for determining new therapeutic targets or determinative biomarkers. A systems biological approach allows comparisons between new models and strengthens predictions for therapeutic pathways. The principal objective of this study was to examine the effects of mutant ATXN2 on genome-wide expression of spinal cord (SC) genes and integrate changes into a framework of previously described transcriptomic changes in ALS models.

Spinocerebellar ataxia type 2 (SCA2) is a dominantly inherited rare polyglutamine disease caused by a CAG repeat expansion in the ataxin-2 (*ATXN2*) gene (1). The SCA2 phenotype includes slow saccadic eye movements and ataxic gait, resulting from progressive failure of cerebellar Purkinje cells, and atrophy of other neuronal structures including the pons. SCA2 is characterized by anticipation where the length of the *ATXN2* repeat is closely tied to disease onset and severity. Unaffected individuals typically have 22 CAGs in *ATXN2*, while repeat lengths ≥33 cause SCA2. Earlier age of onset and greater disease severity are associated with longer CAG repeat expansions which can increase generationally (2). In addition to Mendelian alleles, alleles at the long normal range (>30 repeats) are associated with an increased risk for ALS (3–5). The molecular and genetic association between ATXN2 and ALS may explain the phenotypic overlap observed in SCA2 patients with motor phenotypes characteristically seen in ALS (6).

While there are no disease-modifying treatments for SCA2, preclinical data using *ATXN2* antisense oligonucleotide (ASO) therapeutics are promising. SCA2 is characterized by a gain of toxic function for the polyglutamine expanded ATXN2 protein. We showed that lowering the overall expression of ATXN2 restored phenotypes in two SCA2 mouse models (7). SCA2 mice treated at 8 weeks of age by intracerebroventricular (ICV) injection of an ASO targeting *ATXN2* expression had improved motor, molecular and neurophysiological phenotypes (7).

The importance of ATXN2, however, extends beyond SCA2 and cerebellar neurons. ATXN2 interacts in an RNA-dependent manner with TDP-43, a protein mutated or misfolded in ALS patients. The Gitler Lab showed that the reduction of wild-type *Atxn2* expression in TDP-43 ALS mice either genetically or by an ASO significantly improved mouse survival (8). *ATXN2* intermediate CAG repeat expansions also increase cytoplasmic protein aggregates and motor neuron (MN) dysfunction and death in C9ORF72 ALS patients (9), and frontotemporal dementia (FTD) phenotypes in C9ORF72 ALS patients are modified by intermediated expansions in *ATXN2* (10). These observations place ATXN2 centrally in focus as a potential therapeutic target for ALS as well as FTD.

Despite presence of an ALS-like phenotype in some SCA2 patients, little is known about ATXN2 function in the SC apart from mutant ATXN2 mislocalization in motor neurons in TDP-43 mice (3). Likewise, there have been few studies describing the expression of genes in the ALS SC (11,12). We therefore performed transcriptomic analyses using SCA2 bacterial artificial chromosome (BAC) mice expressing ATXN2 with 72 glutamines driven by a native human *ATXN2* promoter. Analyses included comparisons before and after treatment with *ATXN2* ASO and between SC and cerebellum (CB). Differentially expressed genes (DEGs) in SCA2 SCs included abnormally expressed genes in SCs of ALS patients that may represent therapeutic targets for ALS and some that have potential as ALS or SCA2 biomarkers.

## Results

### SCA2 mice

Both untreated and *ATXN2* ASO7-treated BAC-Q72 mice and wild-type littermates were used in the study. BAC-Q72 mice and ASO7 were described previously (7). The untreated group for which no surgery was performed included 19-week-old BAC-Q72 mice and wild-type littermates. ASO7 treatments were performed using two mouse age groups, designated ‘early’ and ‘late’, referring to age at treatment. Doses averaged 7μg ASO7 per gram of mouse weight (7 mg/kg) in both treatment groups. For the early treatment group, BAC-Q72 or wild-type littermate mice were treated ICV at 8 weeks of age with 175μg of *ATXN2* ASO7 or normal saline, and cerebellar and SC transcriptomes were determined at 19 weeks of age. Mice in the early treatment group were the same as used in our previous study (7). For the late treatment group, BAC-Q72 or wild-type littermate mice were treated ICV at 29 weeks of age with 210μg of *ATXN2* ASO7 or normal saline, and cerebellar and SC transcriptomes were determined at 35 weeks of age. A summary of all mice used in the study, treatment doses and times and group *n* values is provided in Table 1.

**Table 1:** BAC-ATXN2-Q72 mouse groups, ASO treatments and group *n*

Name	ASO dose (7 mg/kg)	Treatment start	Age at sacrifice	Treatment time	Untreated mice		Treated mice			
					WT	TG	WT-SAL	WT-ASO	TG-SAL	TG-ASO
Untreated	−	−	19 weeks	−	4	4	−	−	−	−
Early	175 μg	8 weeks	19 weeks	10 weeks	−	−	4	4	4	4
Late	210 μg	29 weeks	34 weeks	5 weeks	−	−	5	0	3	4
Pooled	175–210 μg	8–29 weeks	19–34 weeks	5–10 weeks	−	−	9	4	7	8

### SCA2 mouse SC transcriptome analyses

Initial analyses characterized DEGs between BAC-Q72 and wild-type SCs from untreated (no saline injection) 19-week-old mice, then in early and late treated mice groups separately and in the pooled group (early pooled with late) of treated mice. Throughout the study, DEGs were defined as those with *AdjP* < 0.05 and a |log2(FC)| ≥ 0.585 (1.5-fold change) (transgenic versus wild-type or transgenic ASO versus transgenic saline). Some DEGs, however, are falling outside of the fold change cutoff but with significant biological relevance are also discussed.

**Figure 1:**
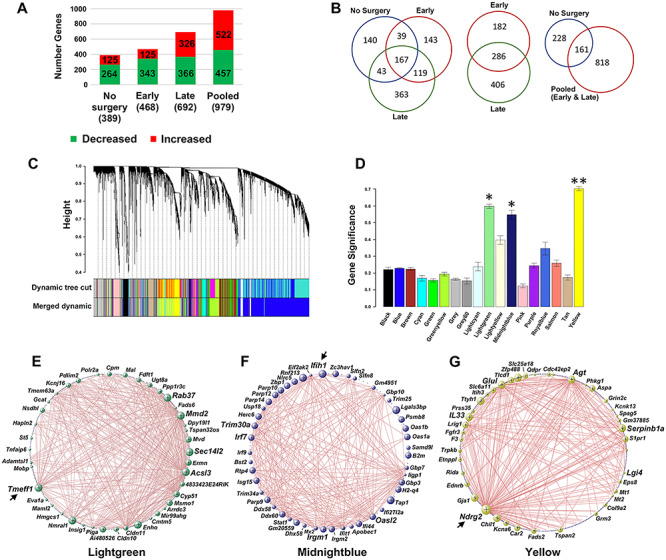
BAC-Q72 SC expression profiles and gene co-expression modules for the pooled dataset. (**A**) Numbers of upregulated or downregulated DEGs in each group and in the pooled set of genes. (**B**) Number of shared DEGs for the untreated group (no surgery), early and late groups. Of all the DEGs in the untreated group, 64% were shared with the combined set of early and late group DEGs, and of all the early and late group DEGs, 32.7% were shared. (**C**) Identification of co-expression modules. Dynamic tree cut analysis gave rise to 18 modules. (**D**) GS per module. The lightgreen, midnightblue and yellow modules were significant (^*^,*P* < 0.05; ^**^, *P* < 0.01). (**EG**) Topological representations of the co-expression networks for the significant modules. The top 40–48 hub genes are shown. Bubble size is proportional to the number of connections. Arrows indicate the top hub gene in each module.

Transcriptome analysis revealed 389 DEGs in SC of the untreated BAC-Q72 mice versus wild-type, 468 DEGs in the early treated group (BAC-Q72 versus wild-type treated with saline) and 692 DEGs in the late group (BAC-Q72 versus wild-type treated with saline) (Fig. 1A). Of the 389 DEGs in the untreated group (BAC-Q72 versus wild-type), 64% (289) were also found in the early and/or the late treatment groups, and 41% (161) were shared with the early and late groups when analyzed as a single pooled group (Fig. 1B). Ranked by *AdjP*, the top 10 DEGs in the early group (*Il33*, *Ndrg2*, *Glul*, *Car2*, *Lrig1*, *Agt*, *Serpinb1a*, *Col9a2*, *Tthy1*, *Atp1a2*) were also among the top 19 DEGs of the late group; all were reduced in expression except *Col9a2* (Supplementary Material, Tables S1–S3). Among the top 10 saline-treated early group genes, 5 were among the top 10 DEGs in the untreated mouse group (*Il33*, *Col9a2*, *Serpinb1a*, *Agt*, *Car2*). The 286 DEGs shared between both the early and late treatment groups represented 61% of the early group DEGs and 41% of the late group DEGs (Fig. 1A and B).

The substantial similarity between the top DEGs in the early and late groups supported pooling the datasets. After pooling, we observed a total of 979 DEGs in SC using the criterion of *AdjP* < 0.05, |log2(FC)| ≥ 0.585, and 161 were shared with the untreated group (Fig. 1A and B). The top 10 DEGs in the pooled group had considerable overlap with the top DEGs in each of the untreated group, early group and late group, including *Il33*, *Agt*, *Glul*, *Ndrg2*, *Lrig1*, *Ttyh1*, *Atp1a2*, *Car2*, *Serpinb1a* and *S1pr1*, all reduced in expression (Supplementary Material, Table S4).

### SCA2 SC genes analyzed by weighted gene co-expression network analysis

**Figure 2:**
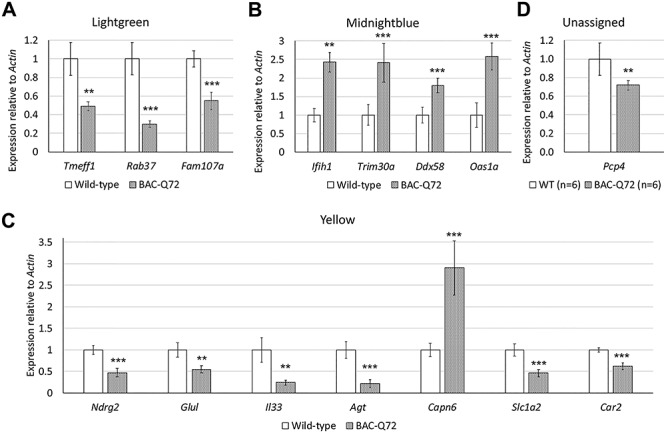
Validation of expression between BAC-Q72 mice and wild-type littermates for the top 2–4 hub genes identified in the lightgreen (**A**), midnightblue (**B**) and yellow (**C**) modules and selected other DEGs determined by qPCR. (**D**) Validation for *Pcp4*, an ALS-related gene, which was not assigned to a module by WGCNA. Values shown are means and SD. All individual wild-type versus BAC-Q72 comparisons were significant at *P* < 0.01. *n* = 5 and 3 mice for the wild-type and BAC-Q72 groups, respectively (A). *n* = 5 and 3 mice for all wild-type and BAC-Q72 groups, respectively, except for *Ddx58* for which *n* = 6 mice per group (B). *n* = 6 mice per group (C and D). Probabilities were determined from unpaired two-tailed Student’s *t*-tests: ^**^, *P* < 0.01; ^***^, *P* < 0.001.

We performed weighted gene co-expression network analysis (WGCNA) to identify relevant gene clusters (co-expression modules) with associated functional pathways, represented by hub gene functional drivers (13). Because of the similarity of the top DEGs between the early and late SC groups, we chose to perform a WGCNA analysis using only the dataset resulting from pooling the early and late groups. Application of hierarchical clustering and dynamic tree cut analysis to the pooled dataset produced 18 modules with 3 that were significant including lightgreen (*P* < 0.05, 488 genes), midnightblue (*P* < 0.05, 148 genes) and yellow (*P* < 0.01, 795 genes) (Fig. 1C and D). Table 2 presents the top 10 DEGs in each of these significant modules. The complete list of gene module membership is in Supplementary Material, Table S5. Network topology analyses for the top 40–48 hub genes are provided illustrating intramodule connectivity (Fig. 1E–G). Validations determined by qPCR were made for the top 2 or 3 genes in the lightgreen, midnightblue and yellow modules and other selected DEGs (Fig. 2).

**Table 2:** Top 10 SC hub genes for the significant modules for BAC-ATXN2-Q72 versus wild-type and Log_2_(FC), ranked by scaled-within values starting with the most interconnected gene (scaled-within = 1)

Lightgreen		Midnightblue		Yellow	
Gene	Log2(FC)	Gene	Log2(FC)	Gene	Log2(FC)
*Tmeff1*	−0.99	*Ifih1*	0.78	*Ndrg2*	−1.3
*Rab37*	−1.5	*Trim30a*	0.53	*Glul*	−1.4
*Acsl3*	−1.01	*Oasl2*	0.60	*Il33*	−2.3
*Mmd2*	−1.1	*Irf7*	0.77	*Serpinb1a*	−2.3
*Sec14l2*	−0.70	*Irgm1*	0.64	*Agt*	−2.8
*Nmral1*	−0.99	*Rtp4*	1.1	*S1pr1*	−1.1
*Enho*	−1.1	*Parp9*	0.81	*Chil1*	−1.6
*Ermn*	−0.99	*Herc6*	0.56	*Gja1*	−1.3
*Cyp51*	−1.3	*Ifit1*	0.60	*Lrig1*	−1.1
*Mvd*	−0.93	*Gbp3*	0.82	*Zfp488*	2.1

### Pathway analyses

We performed pathway analyses by three different methods, including Gene Ontology Enrichment Analysis (GO), Kyoto Encyclopedia of Genes and Genomes (KEGG) analysis and Ingenuity Pathway Analysis (IPA). For each of the analyses, the three approaches are largely agreed. The top pathways for untreated mice, treated mice in the pooled dataset and the three significant modules are shown in Table 3, with complete lists in Supplementary Material, Table S6 and a short list in Supplementary Material, Table S7.

**Table 3:** Top annotated GO, KEGG and IPA pathways in the SC of BAC-ATXN2-Q72 mice versus wild-type and −log_10_(*P*-value) for the indicated comparisons. Benjamini probabilities corrected for multiple pairwise comparisons were calculated for GO and KEGG terms (if significance was not achieved, uncorrected probabilities are shown, indicated by asterisks)

GO		KEGG		IPA	
**TG versus WT (untreated)**					
Glial cell development	5.41	Cholesterol metabolism	3.16	Hepatic fibrosis/hepatic stellate cell activation	5.19
Glial cell differentiation	5.19	Herpes simplex infection	1.65	Interferon signaling	4.74
Ensheathment of neurons	4.57			LXR/RXR activation	4.68
Axon ensheathment	4.57			Th2 pathway	3.99
Gliogenesis	4.57			Th1 and Th2 activation pathway	3.88
Fatty acid biosynthetic process	4.57				
**TG-SAL versus WT-SAL**					
Lipid metabolic process	9.02	Steroid biosynthesis	2.07	Superpathway of cholesterol biosynthesis	9.19
Fatty acid biosynthetic process	5.97	Fatty acid metabolism	1.98	Cholesterol biosynthesis I	7.20
Sterol biosynthetic process	4.82	Biosynthesis of unsaturated fatty acids	1.89	Cholesterol biosynthesis II (via 24,25-dihydrolanosterol)	7.20
Steroid metabolic process	3.63	Biosynthesis of antibiotics	1.85	Cholesterol biosynthesis III (via desmosterol)	7.20
Unsaturated fatty acid biosynthetic process	2.65			Hepatic fibrosis/hepatic stellate cell activation	4.93
**Yellow module**					
Ion transport	4.29^*^	Biosynthesis of unsaturated fatty acids	3.12^*^	Tryptophan degradation X (mammalian via tryptamine)	3.75
Fatty acid biosynthetic process	3.94^*^	Nitrogen metabolism	2.99^*^	Putrescine degradation III	2.85
Aging	3.80^*^	Chemical carcinogenesis	2.41^*^	Th2 pathway	2.76
Adult locomotory behavior	3.42^*^	Neuroactive ligand–receptor interaction	2.25^*^	Oleate biosynthesis II (animals)	2.60
Unsaturated fatty acid biosynthetic process	3.33^*^	Metabolism of xenobiotics by cytochrome P450	2.08^*^	Hepatic fibrosis/hepatic stellate cell activation	2.56
**Lightgreen module**					
Sterol biosynthetic process	5.39	Steroid biosynthesis	3.07	Superpathway of cholesterol biosynthesis	10.50
Lipid metabolic process	2.74	Biosynthesis of antibiotics	1.92	Cholesterol biosynthesis I	8.31
Cholesterol biosynthetic process	2.73	Neuroactive ligand–receptor interaction	1.80	Cholesterol biosynthesis II (via 24,25-dihydrolanosterol)	8.31
Steroid metabolic process	2.63			Cholesterol biosynthesis III (via desmosterol)	8.31
Steroid biosynthetic process	2.18			Zymosterol biosynthesis	4.46
**Midnightblue module**					
Defense response to virus	26.81	Herpes simplex infection	7.49	Interferon signaling	13.20
Immune system process	22.69	Influenza A	6.49	Activation of IRF by cytosolic pattern recognition receptors	12.40
Innate immune response	19.78	Measles	6.51	Antigen presentation pathway	7.63
Response to virus	17.48	Hepatitis C	5.47	Role of RIG1-like receptors in antiviral innate immunity	5.70
Cellular response to interferon-beta	17.16	RIG-I-like receptor signaling pathway	2.94	Role of pattern recognition receptors in recognition of bacteria and viruses	5.40

Several significant pathways were annotated for the untreated mouse group (BAC-Q72 versus wild-type). The top pathways and their DEGs included glial cell growth, neuron ensheathment, fatty acid synthesis, cholesterol metabolism, herpes simplex, interferon signaling, hepatic stellate cell activation, LXR/RXR signaling and Th1/Th2 immune response signaling. Transgenic mice in the pooled group that were treated with saline had pathways in lipid metabolic processes and steroid/cholesterol biosynthesis most significant and hepatic stellate cell activation like for the untreated mouse group.

When evaluated by module, ion transport, fatty acid biosynthesis and hepatic stellate cell activation annotations were represented in the yellow module. DEGs in the yellow module involved in ion transport included potassium channels, glutamate receptor and glutamate synthesis genes, calcium channels, chloride channels, mechanotransducer channels and other ion transporters. Other top pathways annotated in this module were fatty acid synthesis, hepatic stellate cell activation and Th2 pathway activation. The lightgreen module was predominantly characterized by DEGs regulating steroid and cholesterol biosynthesis. The midnightblue module was dominated by DEGs functioning in innate immunity, with upregulation of all DEGs.

**Table 4:** Significant DEGs in the SC of BAC-ATXN2-Q72 mice treated with ASO7 versus saline, ranked by significance

Gene	Log2(FC)	AdjP	Footnote
*Ctss*	0.74	0.0000075	2
*Mmp12*	1.4	0.000011	
*Gpnmb*	1.3	0.000038	
*C1qa*	0.69	0.00012	1
*Trem2*	0.74	0.00018	1,3
*Cd300c2*	0.74	0.00018	
*Mpeg1*	0.82	0.00024	
*Hvcn1*	0.79	0.00053	
*C4b*	0.67	0.0013	1,3,4
*Gm23969*	1.1	0.0026	
*Tyrobp*	0.60	0.0097	1
*Steap1*	−1.0	0.010	
*Lgals3*	0.98	0.020	1
*Atp6v0d2*	0.95	0.021	2
*C3*	0.86	0.022	1,2,3,4
*Gm6166*	0.77	0.022	
*Clic6*	−0.94	0.023	
*Rgs1*	0.98	0.025	
*Ifi202b*	0.95	0.031	1
*Cd68*	0.80	0.031	2
*Clec7a*	0.80	0.031	1,2
*Sspo*	0.64	0.037	
*Il1rrn*	0.93	0.049	1,4
*Adgrg5*	0.90	0.049	

1, Innate immunity. 2, Phagosome/Lysosome. 3, Complement component. 4, LXR/RXR, FXR/RXR activation.

### Effect of ASO7 on DEGs

**Figure 3:**
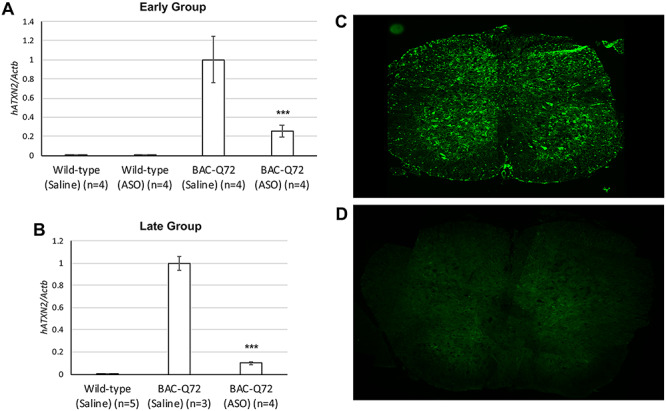
ASO7 uptake and reduction of *ATXN2* in SCA2 mouse SC. (**A** and **B**) Validation of ASO7 inhibition of *ATXN2* in SC of BAC-Q72 by qPCR for both the early (A) and late (B) groups. Values shown are means ± SD. (**C** and **D**) Anti-ASO antibody labeling in SCA2 mouse thoracic SC following ASO7 injection (C) and saline injection (D), determined by immunofluorescent labeling. Probabilities were determined from unpaired two-tailed Student’s *t*-tests between the BAC-Q72 saline groups and the BAC-Q72 ASO groups: ^***^, *P* < 0.001.

Previously, we demonstrated that SCA2 mice treated with ASO7 had improved motor, molecular and neurophysiological phenotypes. Reduction of *ATXN2* expression genetically or by using an ASO therapeutically increased survival of TDP-43 ALS mice (7,8). Therefore, we wanted to characterize the SC transcriptome in SCA2 mice after ASO7 treatment. The expression of *ATXN2* was significantly reduced in SCA2 mice treated ICV with ASO7, with 25 and 10% *ATXN2* expression remaining for the early and late groups, respectively (Fig. 3A and B). Immunofluorescent labeling of SC from selected ASO7-treated mice using an anti-ASO antibody revealed ASO uptake in cells consistent with astrocytes and motor neurons (Fig. 3C and D). WGCNA analysis of ASO-treated mice using the pooled dataset demonstrated 18 modules but none reached our pre-specified significance levels. There were 57 genes with significant *AdjP* values (<0.05), and 24 DEGs occurred when using the cutoff criterion of |log2(FC)| ≥ 0.585. A list of these DEGs including log2(FC) and *AdjP* values is provided in Table 4. Pathway analysis showed that ASO7 treatment modified innate immunity and defense pathways, LXR/RXR and FXR/RXR activation and phagosome or lysosomal maturation (Table 5; Supplementary Material, Table S7). ASO7 treatment reversed the direction of dysregulation (different log2(FC) sign) in 16 of 57 significantly different genes. These 16 genes are *Fyco1*, *D1Ertd622e*, *Tsc22d4*, *Gpt*, *Steap1*, *Lgmn*, *Tst*, *Pfdn4*, *C3*, *Gm6166*, *Clic6*, *Pcdhga4*, *Tbcd*, *Wdr53*, *Slc8a3 and Arrdc3*, ranked in order of *AdjP* for the ASO7 versus saline comparison (Supplementary Material, Table S8).

**Table 5:** All significant GO, KEGG and IPA pathways in the SC of ASO7-treated BAC-ATXN2-Q72 mice versus saline and −log_10_(*P*-value). Benjamini probabilities corrected for multiple pairwise comparisons are shown for GO and KEGG terms

GO		KEGG		IPA	
Innate immune response	4.66	*Staphylococcus aureus* infection	1.87	Complement system	5.21
		Tuberculosis	1.85	LXR/RXR activation	3.66
		Complement and coagulation cascades	1.73	FXR/RXR activation	3.61
		Pertussis	1.66	Role of pattern recognition receptors in recognition of bacteria and viruses	3.50
		Phagosome	1.56	Acute phase response signaling	3.23
		Lysosome	1.41	Dendritic cell maturation	3.07
		Systemic lupus erythematosus	1.32	Crosstalk between dendritic cells and natural killer cells	2.47
				Phagosome maturation	2.04

As ASO treatments may activate innate immunity pathways, we also compared wild-type mice treated with ASO7 versus wild-type mice treated with saline and observed only six DEGs using the criterion of AdjP<0.05 and a |log2(FC)| ≥ 0.585, none of which functioned in pathways of immunity (Supplementary Material, Table S9). Mice used for this comparison were those in the pooled group shown in Table 1.

**Figure 4:**
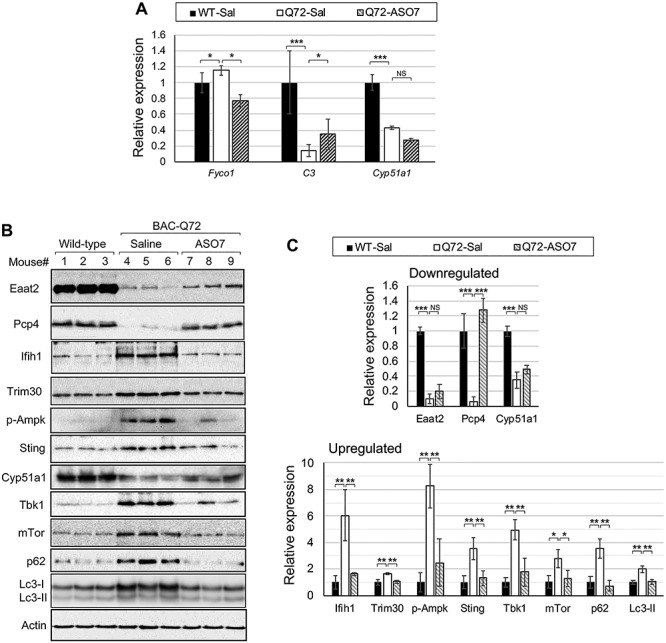
Restoration of selected genes in SC of BAC-Q72 mice with ASO7 treatment. (**A**) Effect of ASO7 on *Fyco1*, *C3* and *Cyp51a1*. Expression determined by qPCR relative to *Gapdh*. Reduced expression of *C3* is significantly increased by ASO7, while increased expression of *FycoI* is significantly decreased by ASO7. Reduced *Cyp51a1* expression was not improved by ASO7. (**B**) Effect of ASO7 on the expression of Eaat2 (Slc1a2), Pcp4, Ifih1, Trim30, p-Ampk, Sting, Cyp51a1, Tbk1, mTor, p62 and Lc3 determined by western blotting. (**C)** Densitometric quantifications of western blots (*n* = 3–5 mice). Bonferroni corrected Student’s *t*-tests. NS, not significant; ^*^, *P* < 0.05; ^**^, *P* < 0.01; ^***^, *P* < 0.001.

*Validation of expression following ASO7 treatment.* A selection of DEGs in the SC transcriptomes, as well as selected other genes suspected to be abnormally expressed based on pathway analyses, were investigated by qPCR and western blotting following ASO7 treatment. *Fyco1*, the most significantly reversed DEG in the transcriptome data following ASO7 treatment (see above), was also significantly increased in SCA2 mice following ASO7 treatment by qPCR (Fig. 4A). Similarly, *C3* was significantly reduced in SCA2 mouse SC and significantly increased following ASO7 treatment (Fig. 4A). *Cyp51a1* was investigated by qPCR as an example of DEGs regulated downstream of SREBP. It was significantly reduced but ASO7 had no effect on improving its expression (Fig. 4A). We also validated several proteins by western blotting, because protein changes can sometimes be more readily observed versus mRNA changes following ASO7 treatment (7). Proteins that we investigated by western blotting included Eaat2, Pcp4, Ifih1, Trim30, p-Ampk, Sting, Cyp51a1, Tbk1, mTor, p62/Sqstm1 and Lc3-II. Of these, all were significantly increased or decreased in SCA2 mouse SC (Fig. 4B and C). ASO7 treatment significantly reversed the expression of all except for Eaat2 and Cyp51a1 (Fig. 4B and C).

### ALS genes

ALS signaling was a significant pathway annotated by IPA of BAC-Q72 mice (Supplementary Material, Table S6). In SC of untreated mice, the annotation consisted of *Fgfr3*, *Fgfr2*, *Glul*, *Slc1a2*, *Grin2C* and *Capn11*. In the yellow module, the ALS annotation included *Fgfr2*, *Fgfr3*, *Glul*, *Slc1a2 and Grin2c*. The ALS signaling pathway was also annotated in CB of BAC-Q72 mice, represented by *Glul*, *Pik3cd*, *Capn5*, *Gria1*, *Capn6*, *Cacna1s*, *Grid2*, *Slc1a2*, *Kl* and *Bid*. Western blots for the ALS-related proteins Eaat2, Pcp4 and Tbk1 are discussed immediately above and presented in Figure 4B and C. Apart from the IPA-annotated pathway, other ALS-associated genes were differentially expressed, described in the Discussion section below.

### Cerebellar genes

Comparison of significant DEGs in SC to those in the same mice in the CB revealed substantial overlap. Of 824 DEGs found in the CB (early and late mice pooled, BAC-Q72 versus wild-type, saline treated), 202 were also differentially expressed in the SC. GO, KEGG and IPA pathway analyses of the overlapping genes revealed similarity to the SC midnightblue module, with top annotations including genes functioning in innate immunity, while cholesterol biosynthesis, characterizing the SC lightgreen module, was also represented by IPA (Table 6). A table of DEGs in the CB is provided in Supplementary Material, Table S10, and a list of overlapping genes with a graphic representation of the *AdjP* values is provided in Supplementary Material, Figure S1. The output from pathway analyses is provided in Supplementary Material, Table S6. Of the cerebellar DEGs in our previous study (14) defined by the same criteria used in this study (AdjP<0.05 and a |log2(FC)| ≥ 0.585), 17% (138 DEGs) were observed differentially expressed in the CB in this study (Supplementary Material, Table S11). The relatively low overlap likely reflects a disparity in mouse ages (8 weeks in the previous study), yet pathway analyses demonstrated shared annotations, including many innate immunity genes at the top of the KEGG and GO lists (Supplementary Material, Table S11).

**Table 6:** Top annotated GO, KEGG and IPA pathways in the CB of BAC-ATXN2-Q72 mice versus wild-type, and shared between CB and SC, and -log_10_(*P*-value). Benjamini probabilities corrected for multiple pairwise comparisons were calculated for GO and KEGG terms

GO		KEGG		IPA	
**CB: TG versus WT**					
Immune system process	11.83	Viral myocarditis	2.12	Interferon signaling	4.97
Defense response to virus	9.48	Measles	1.98	Antigen presentation pathway	4.79
Response to virus	5.75	Herpes simplex infection	1.97	Role of pattern recognition receptors in recognition of bacteria and viruses	4.68
Cellular response to interferon-beta	5.44	Cell adhesion molecules (CAMs)	1.94	Virus entry via endocytic pathways	4.16
Innate immune response	4.92	Influenza A	1.31	Glutamate receptor signaling	3.48
Negative regulation of viral genome replication	3.52			Th2 pathway	3.13
Cell adhesion	3.40			Complement system	3.07
Ion transport	2.32			Caveolar-mediated endocytosis signaling	2.83
**SC CB shared**					
Defense response to virus	8.44	Measles	2.58	Role of pattern recognition receptors in recognition of bacteria and viruses	4.67
Immune system process	4.45	Hepatitis C	2.34	Interferon signaling	3.63
Response to virus	4.38	Influenza A	2.28	Role of RIG1-like receptors in antiviral innate immunity	3.29
Innate immune response	2.90	Herpes simplex infection	2.16	Neuroprotective role of THOP1 in Alzheimer’s disease	3.28
Cellular response to interferon-beta	2.14			Hepatic fibrosis/hepatic stellate cell activation	2.97
				Superpathway of cholesterol biosynthesis	2.78
				Retinoic acid mediated apoptosis signaling	2.73
				Activation of IRF by cytosolic pattern recognition receptors	2.7
				Acute phase response signaling	2.49
				LXR/RXR activation	2.44
				Antigen presentation pathway	2.4

### Novel biomarker predictions

To predict potential CSF biomarkers for ALS, we determined the overlap of SCA2 mouse SC DEGs with the list of predicted secreted proteins in the Human Protein Atlas (available from www.proteinatlas.org) (15). A complete list is presented in Supplementary Material, Table S12.

## Discussion

We developed a systems approach to understand the SC pathology related to mutant *ATXN2*, enabled by *ATXN2* expression in the SC of SCA2 BAC-Q72 mice. In addition to cerebellar disease, lower motor neuron dysfunction is detected in 12% of SCA2 cases (16). The objective of this study was to use this mouse model to understand genome-wide expression changes in the SC. We also determined those networks responsive to *ATXN2* ASO treatment to further delineate mechanisms underlying SCA2 and ALS and to reveal pathways and targets that might be exploited therapeutically.

### ALS-associated genes

The SCA2 mouse SC transcriptome reflects the ALS transcriptome at multiple levels. Several DEGs found in two human ALS SC transcriptome studies also appeared in this study (Supplementary Material, Fig. S1), including DEGs in innate immunity, LXR and lipid metabolism pathways (11,12). In a comprehensive review of ALS literature (17), 10 genes (Supplementary Material, Table S7) were linked to the progression of ALS, among which were 3 (*Slc14a1*, *Pcp4 and Aqp4*) that were also DEGs in SCA2 mouse SC. Mouse studies supported our findings as well: transcriptome analysis of SC tissues from *TDP-43* transgenic mice (18) identified 30 DEGs among which 5 (*Serpina3n*, *Cst3*, *Nefl*, *Nefh*, *Scd1*) were also DEGs in SC of SCA2 mice. Despite that SOD1 mice lack TDP-43 proteinopathy, we also compared DEGs in SCA2 and SOD1 mice. A total of 9 DEGs overlapped with the 52 found in SC of presymptomatic SOD1 mice (19) and another subset of 9 DEGs with the 126 genes in the ALS Online Database (ALSoD) (20) (Supplementary Material, Table S7).

Since glutamate toxicity is a hallmark of ALS, we found it notable that various glutamate transporters and receptors were significantly reduced in SCA2 mouse SC. Genes included *Grm3* encoding metabotropic glutamate receptor 3, *Grin2c* encoding glutamate ionotropic receptor NMDA type subunit 2C, *Glul* encoding glutamine synthetase and *Slc1a2* encoding the glial excitatory amino acid transporter 2 (EAAT2) required for synaptic glutamate reuptake. Also, the regulator of G protein signaling 8 gene *Rgs8* was abnormally reduced in SCA2 mouse CB (this study and previously (14)), consistent with our observation of enhanced mGluR1-mediated excitatory postsynaptic currents (EPSCs) and elevated calcium in SCA2 Purkinje neurons (21). Rgs1, Rgs4 and Rgs16 were also differentially expressed in SCA2 mouse SC.

Poly(ADP-ribose) polymerases (PARPs) are associated with ALS-related accumulation of TDP-43, and targeting PARP-1/PARP-2 with small molecules resulted in normalization of TDP-43 aggregates in a fly model of ALS (22). We found multiple PARPs significantly increased in the SCA2 mouse transcriptomes, including PARPs 4, 8, 9, 12 and 14 in SC and 9, 10, 12 and 14 in CB. Like PARP-1/PARP-2, PARP-12 and PARP-14 have roles in stress granule (SG) assembly (23).

### Three pathways converging at the endoplasmic reticulum

**Figure 5:**
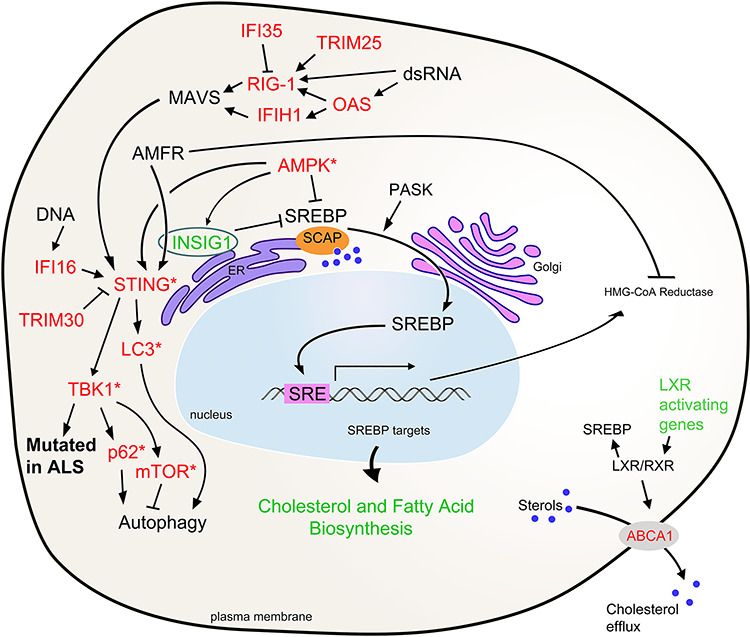
Highly interconnected pathways altered in SCA2 mouse SC transcriptome converge at the ER. Regulation of the innate immunity pathway by STING and the cholesterol and fatty acid biosynthesis pathways by SREBP is dependent on INSIG1 and polyubiquitination by AMFR anchoring STING to the ER membrane. Innate immunity can be activated by dsRNAs produced by ER stress and activation of the RIDD pathway or DNAs from damaged mitochondria. STING activation can directly modify LC3 and can activate TBK1, p62 and mTOR to regulate autophagy. INSIG1 anchors SCAP and SREBP to the ER when sterols are abundant. When sterols are low, SCAP and SERBP translocate to the Golgi via COPII vesicles in a PASK-dependent manner where SREBP is processed and then its bHLH domain fragment is translocated to the nucleus to activate cholesterol and fatty acid biosynthesis genes. Among these are HMG-CoA reductase which catalyzes the rate limiting step in cholesterol biosynthesis, is the target of statins and is inhibited by AMFR polyubiquitination. We also observed genes activating LXR pathways reduced in SCA2 mouse SC transcriptomes. Finally, Ampk is activated in SCA2 mouse SC, which inhibits SREBP and activates STING. Green represents genes downregulated and red upregulated in SCA2. Asterisks indicate upregulated proteins from nonsignificant DEGs.

An exciting finding in this study is the elevated expression of numerous genes in the innate immunity pathway regulated by STING and the mostly downregulated genes controlled by SREBP in SCA2 mouse SC that regulate cholesterol and fatty acid biosynthesis. Consistent with this, we found that Ampk which is an activator of STING and inhibitor of SREBP was highly activated in SCA2 SC. Many genes in these interconnected pathways were observed previously in human ALS SC, discussed in the following paragraphs. An illustration of these pathways with indication of the relevant DEGs and proteins is provided in Figure 5.

*Innate Immunity.* The midnightblue module was characterized by upregulated DEGs functioning in innate immunity, many of which are shared with human ALS (11,12). Among these were the retinoic acid-inducible gene 1 (RIG-1)-like receptors (RLRs) *Ddx58* and *Ifih1*. *Ddx58* encodes RIG-1 and *Ifih1* encodes melanoma differentiation-associated protein 5 (MDA5) and was the top midnightblue hub gene. RIG-1 and MDA5 function together with mammalian 2′-5′-oligoadenylate synthase (OAS) genes, of which we observed four upregulated in SC and seven in CB (Supplementary Material, Table S7). RLRs and OASs are activated by dsRNAs that can originate by various causes (24–26) including activation of regulated IRE1-dependent decay (RIDD) following endoplasmic reticulum (ER) stress (27,28). Downstream, RLRs and MDA5 activate MAVS on the mitochondrial membrane, leading to STING activation at the ER (29,30). The activation of STING requires it to be polyubiquitinated by AMFR (GP78) to anchor it on the ER membrane and is dependent on INSIG1 (31) that was downregulated in SCA2 mouse SC. STING activation leads to TBK1 activation of interferon regulatory factors (IRFs) (32,33), including *Irf7* that was a top 10 midnightblue hub gene. *Trim30a*, a major STING inhibitor (34), was also among this top 10. Unexpectedly, none of the IRF-responsive genes, those with the interferon-stimulated response element (ISRE), were observed in SCA2 mouse transcriptomes.

*Innate immunity and a link to autophagy.* Once we determined that pathways leading to STING activation were upregulated in SCA2 mouse transcriptomes without activation of ISRE genes, we investigated Tbk1 and autophagy downstream. We had previously observed abnormal autophagosome production associated with increased abundance of the SG protein Staufen1 with ATXN2 mutation (35), and TBK1 supports autophagosome formation (36). Also, *TBK1* loss of function mutations are observed in ALS and FTD (37). We investigated both Tbk1 and its substrates mTor and p62/Sqstm1, and the autophagy marker Lc3-II, finding that the protein abundances of each were increased in SCA2 mice but restored by ASO7 (Fig. 4B and C). STING regulation of autophagy following innate immunity activation has been observed previously and involves STING-LC3 direct interaction but can also be TBK1-dependent (38–40) (Fig. 5). In addition, the most downregulated DEG, Il33, in our analysis is neuroprotective against disease-related autophagy (41,42).

*Fatty acid biosynthesis:* Fatty acid synthesis was an annotated pathway of our yellow module, including nine DEGs (Supplementary Material, Table S7). *Acsl3* was also significantly reduced in the lightgreen module, encoding long-chain acyl-CoA synthetase that directs acyl chains into lipid droplets (43). Altered fatty acid synthesis in SCA2 mouse SC is likely explained by changes in mTOR abundance downstream of ataxin-2: MEFs deficient for ataxin-2 had elevated mTOR (44), which promotes fatty acid synthesis by activating SREBP (45). Insig1, which controls fatty acid and cholesterol synthesis, was also abnormally expressed in *Atxn2* knockout mice (46). Consistent with this, abnormal fatty acid synthesis was observed in the liver of *Atxn2* knockout mice and SC of ATXN2-CAG100 mice (47,48), cellular fat content was increased in *Caenorhabditis elegans* null for *Atxn2* (49), and *Atxn2* knockout mice develop obesity (44,50).

*Cholesterol biosynthesis.* Cholesterol cannot pass the blood–brain barrier; thus the brain must synthesize its own which is done in astrocytes, and when cholesterol synthesis is lost, neuronal dysfunction is the result (51). The lightgreen module was characterized by 12 DEGs in the cholesterol biosynthesis pathway, a significant annotated pathway in human ALS (11). A key regulator of this pathway is SREBP that in times of high cholesterol is sequestered to the ER by INSIG1 and the SREBP cleavage activating protein SCAP. When cholesterol is reduced, INSIG1 and SCAP uncouple allowing SREBP processing mediated by SCAP and translocation to the nucleus where it activates cholesterol synthesis genes (52). SREBP nuclear translocation is also dependent on PAS kinase (PASK) (53), which phosphorylates the ATXN2 homolog Pbp1 in yeast (54). Described above, INSIG1 also regulates STING in innate immunity. Innate immunity is coupled to cholesterol synthesis whereby low cholesterol is associated with elevated innate immunity signaling and vice versa (55,56). Genes activating LXR/RXR signaling were also reduced in SCA2 mouse SC as well as in human ALS (11); LXR variants are associated with increased ALS risk (57). LXRs also regulate cellular efflux of cholesterol, which when restored in SCA2 or SCA3 mice improved autophagy and protein aggregation phenotypes (58).

### ASO treatment

Improved pathology of TDP-43 and SCA2 mice after lowering Atxn2 expression (7,8) was a motivation for our characterizing genes in SCA2 mice altered by *ATXN2* ASO7 treatment. Many of the genes altered by ASO7 treatment function in the same pathways that characterize SCA2 mouse SC (Table 4). Among these, it is notable that *Lgals3bp* was increased in SCA2 mouse SC while the gene for its binding partner *Lgals3* (encoding galectin-3) was increased in SC by ASO7 treatment. Both have innate immunity functions. Differentially expressed complement component genes *C4b* and *C3* function in innate immunity, LXR/RXR signaling and lipid and cholesterol metabolism (59). For some abnormally expressed DEGs in SCA2 mouse SC, ASO7 treatment could partially or fully restore expression. For *C3*, its expression in SCA2 mouse SC was partially restored by ASO7 treatment (Fig. 4A). The most significant DEG in ASO7-treated SCA2 SC was *Ctss*, encoding cathepsin S, whose expression is also stimulated by innate immunity activation (60), and promotes autophagy (61). Another DEG upregulated by ASO7 was *Clec7a*, encoding Dectin-1, that traffics autophagic vesicles with its interacting partner Fyco1 (62), which was altered in SCA2 SC and restored by ASO7 treatment (Fig. 4A). *Pcp4* which is ALS-associated was significantly reduced in SCA2 mouse SC and restored by ASO7 treatment (Fig. 4B and C). ASO7 also partially restored EAAT2 levels (Fig. 4B and C). IFIH1 and TRIM30 were also restored by ASO treatment (Fig. 4B and C). Notably, there were only six DEGs in SC of wild-type mice treated with ASO7, supporting that lowering ATXN2 expression therapeutically would be well tolerated with little off-target effects or inflammation.

### Biomarkers

Some DEGs observed in SCA2 mouse SC encoding small or circulated peptides or processed proteins merit investigation as biomarkers for ALS. *Il33* was the most highly downregulated gene in SCA2 mouse SC, consistent with a study that found reduced IL33 in cerebrospinal fluid (CSF) from ALS patients (63). Recently, three chitinases were reported upregulated in ALS patient CSF suggesting their use as biomarkers (64). Two chitinase genes were differentially expressed in SCA2 mouse SC, including *Chil1* (downregulated) and *Chia1* (upregulated). Lastly, *NEFL* and *NEFH* were both significantly downregulated in human ALS SC (11) and SCA2 mouse SC. Previous studies showed that both NEFL and NEFH are promising CSF or serum biomarkers that are elevated as motor neurons are lost (65,66).

## Conclusions

Our study establishes the importance of ATXN2 in motor neuron disease and SCA2 mouse models. Expression of mutant ATXN2 in the SC produced a set of DEGs that was shared with previous transcriptome analyses of human ALS SC, TDP-43 mouse SC and SOD1 mouse SC and a set of DEGs that were altered by *ATXN2* ASO therapy. While relatively few individual DEGs were shared among the different models, there was significant overlap in dysregulated pathways characterizing SCA2, TDP-43 and SOD1 mice. This observation also extended to dysregulated pathways in SC of ALS patients. DEGs revealed in this study may represent therapeutic targets for ALS or lead to biomarkers useful for characterizing ALS therapeutics. The similarity of pathways in SCA2 mouse SC compared with human ALS SC indicates that the SCA2 mouse may be valuable for evaluating experimental therapeutics for ALS.

## Materials and Methods

### SCA2 mice

BAC-ATXN2-Q72 (BAC-Q72) mice were previously described (7,14). BAC-Q72 are transgenic for the complete human *ATXN2* gene with all introns and exons, including 16 kb upstream sequence driving *ATXN2* expression and the complete 3'-UTR. The mice used in this study were maintained on a mixed B6;D2 background with backcrossing to wild-type vendor-purchased (Jackson Laboratories) mice every five generations. Mouse husbandry and surgical procedures were in accordance to the Institutional Animal Care and Use Committee (IACUC)-approved protocols.

### ASO treatment of SCA2 mice

Mice were treated with ASO7 or normal saline in two separate experiments. In the first experiment (early treatment group), BAC-Q72 mice or wild-type littermates were treated at 8 weeks of age and sacrificed at 19 weeks of age (10 weeks treatment time) and are a subset of the same mice that appear in Figure 1B of our previous study (14). In the second experiment (late treatment group), BAC-Q72 mice or wild-type littermates were treated at 29 weeks of age and were sacrificed at 34 weeks of age (5 weeks treatment time). Treatments were made by ICV injection of mice anesthetized with a mixture of oxygen and isoflurane using a Hamilton 26-s gauge needle and a Stoelting stereotaxic frame. Injections consisted of 6 μL of 35 μg/μl ASO7 diluted in normal saline for a total of 210 μg. Control mice received the same volume of normal saline. Anesthesia was initiated using 3% isoflurane for 5 min, and the isoflurane mixture was lowered to 2% during injections. Stereotaxic bregma coordinates were −0.46 mm anteroposterior, −1.0 mm lateral (right side) and −2.5 mm dorsoventral. Needles were removed 4 min after ASO delivery. Mice were maintained on a 39°C isothermal pad while anaesthetized and during recovery. After treatment, mice were sacrificed and SCs were removed and split lengthwise with half used for RNA preparations for RNA-seq and quantitative real-time PCR (qPCR) and the remaining half used for western blotting.

### RNA preparation

Total RNA was extracted from tissues using the RNaeasy Mini-Kit (Qiagen Inc.) according to the manufacturer’s protocol. DNAse I-treated RNAs were used to synthesize cDNA using the ProtoScript cDNA First Strand cDNA Synthesis Kit (New England Biolabs Inc.). RNA quality was determined using the Bioanalyzer2100 Pico Chip (Agilent). Samples with an RNA integrity number (RIN) > 8 were considered with acceptable quality. Prepared RNAs were used for RNA-seq and qPCR as described below.

### RNA-seq

Library preparation was performed using the Illumina TruSeq Stranded Total RNA Sample Prep and Ribo-Zero rRNA Removal Kit for mouse. Single-end 50-bp reads were generated on a Hiseq 2000 sequencing instrument at the University of Utah Microarray and Genomic Analysis Shared Resource using Illumina Version 4 flow cells. Reads were then aligned to the mouse reference genome (mm10) by Novoalign (http://www.novocraft.com). Quality of RNA sequencing was considered acceptable with an average of 22 million reads. After read alignment, DEGs were identified using the DRDS application (version 1.3.0) in the USeq software package (http://useq.sourceforge.net/).

### Co-expression network

RNA-seq data were first filtered by FPKM (≥1.0 in 90% of samples) to reduce noise. Genes were ranked by coefficient of variation (≥0.15) and the top 10 000 most variable genes were used for the analysis. The unsigned WGCNA was conducted as previously described using the R package WGCNA (67). Briefly, a similarity matrix was constructed using the Pearson correlation coefficients created between the FPKM normalized expression levels of the input transcripts. Raising the absolute value of the Pearson correlation coefficients to a power of 10 produced a scale-free topology index above 0.9, resulting in a network with few, large correlations at the expense of lowly correlated transcripts. This allows for the fewer, highly connected and biologically relevant hub genes. An adjacency network was then created using topological overlap measure (TOM), a measure of neighborhood connectivity. To create modules, the adjacency network was converted into a dissimilarity measure (1 − TOM) and clustered using flashClust, a hierarchical clustering function. Cluster branches were cut to identify modules. Module size was set to a minimum of 50 transcripts, and modules with a 5% similarity were merged using dynamic tree cutting. To identify significant modules, gene significance (GS) was calculated as the absolute value of the correlation between gene expression and transgenic status. Overall significance for each module was calculated by averaging all GS within each module. Statistical significance was determined by using a *t*-test for correlation.

### Functional enrichment analysis

Gene Ontology Enrichment Analysis (GO), and Kyoto Ency-clopedia of Genes and Genomes (KEGG) analysis was conducted using the functional annotation tool DAVID (https://david.ncifcrf.gov/). Ingenuity pathway analysis (IPA) was performed using the IPA software package (Qiagen). Enriched ontological terms and pathways with *P*-values less than 0.05 were selected.

### Quantitative real-time PCR (qPCR)

Quantitative real-time PCR (qPCR) was performed using the QuantStudio 12K PCR System (Life Technologies, Inc.) with the Power SYBR Green PCR master mix (Applied Biosystems Inc.) or FastLane Cell SYBR Green Kit reagents. PCR amplification was carried out for 45 cycles. Cycling parameters were denaturation (95°C for 10 s), annealing (60°C for 10 s) and extension (72°C for 40 s). The threshold cycle for each sample was chosen from the linear range and converted to a starting quantity by interpolation from a standard curve run on the same plate for each set of primers. All primers used in the study are presented in Supplementary Material, Table S13.

### Western blot assays

SC protein extracts were prepared by homogenization of mouse SC in extraction buffer [25 mM Tris-HCl pH 7.6, 300 mM NaCl, 0.5% Nonidet P-40, 2 mM EDTA, 2 mM MgCl_2_, 0.5 M urea and protease inhibitors (Sigma-Aldrich, P8340)] followed by centrifugation at 4°C for 20 min at 14 000 RPM. Supernatants only were used for western blotting. Protein extracts were resolved by SDS–PAGE and transferred to Hybond (Amersham) followed by detection with ECL reagent (Amersham). Antibodies included IFIH1 rabbit polyclonal antibody (1:3000, Proteintech Cat# 21775–1-AP), TRIM30 antibody (1:3000, Novus Biologicals, NBP2-41087), Anti-PCP4 antibody (1:5000, Abcam, ab197377), SQSTM1/p62 antibody (1:4000, Cell Signaling, Cat# 5114), mTOR antibody (1:4000, Cell Signaling, Cat# 2972), LC3B antibody (1:7000, Novus Biologicals, NB100-2220), GLT-1/SLC1A2/EAAT2 antibody (9 HCLC), ABfinity™ Rabbit Oligoclonal (1:3000, Thermo Fisher Scientific, Cat#: 711020) and monoclonal anti-β-Actin−peroxidase antibody, clone AC-15 (1:20 000, Sigma-Aldrich, A3854). The secondary antibody was peroxidase-conjugated AffiniPure Goat Anti-Rabbit IgG (H + L) antibody (1:5000) (Jackson ImmunoResearch Laboratories, Cat# 111–035-144).

### Immunofluorescent labeling

SC tissue was excised and processed in 4% paraformaldehyde for 72 h and then sequentially treated in 10, 20 and then 30% sucrose/phosphate-buffered saline (PBS), 24 hours each. The tissue was then frozen mounted in OCT and 18μm sectioned coronally on a cryostat. Sections were permeablized in PBS, 0.5% Triton X-100, for 10 min and then washed in wash buffer (PBS, 0.05% Triton X-100). Sections were blocked in diluent buffer (PBS, 0.05% Triton X-100, 3% normal goat serum) for 5 h. Primary rabbit anti-ASO antibody (1:10 000, provided by Ionis Pharmaceuticals) in diluent buffer was incubated on tissues overnight at 4°C. Cells were washed then incubated with secondary goat anti-rabbit Alexa Fluor 488 (Jackson ImmunoResearch 111-545-003) in diluent buffer for 2 h at room temperature. Cells were washed and mounted using Fluoro-Gel (Electron Microscopy Sciences). Fluorescent images were collected using a Nikon C1 fluorescent microscope with a 488 nm filter and a 408/488/561 dichroic mirror.

## Supplementary Material

Supplementary_Figure_1_ddaa072

Supplementary_Figure_2_ddaa072

Supplementary_Table_1_ddaa072

Supplementary_Table_2_ddaa072

Supplementary_Table_3_ddaa072

Supplementary_Table_4_ddaa072

Supplementary_Table_5_ddaa072

Supplementary_Table_6_ddaa072

Supplementary_Table_7_ddaa072

Supplementary_Table_8_ddaa072

Supplementary_Table_9_ddaa072

Supplementary_Table_10_ddaa072

Supplementary_Table_11_ddaa072

Supplementary_Table_12_ddaa072

Supplementary_Table_13_ddaa072

Legends_to_Supplemtary_Tables_ddaa072
