# Supplementary material for: ALS-associated genes in SCA2 mouse spinal cord transcriptomes
Source: Hum Mol Genet. 2020 Apr 20;29(10):1658–72. doi: 10.1093/hmg/ddaa072 (PMC7322574; doi:10.1093/hmg/ddaa072)
Supplement: Supplementary_Figure_1_ddaa072 [file supplementary_figure_1_ddaa072.pdf]

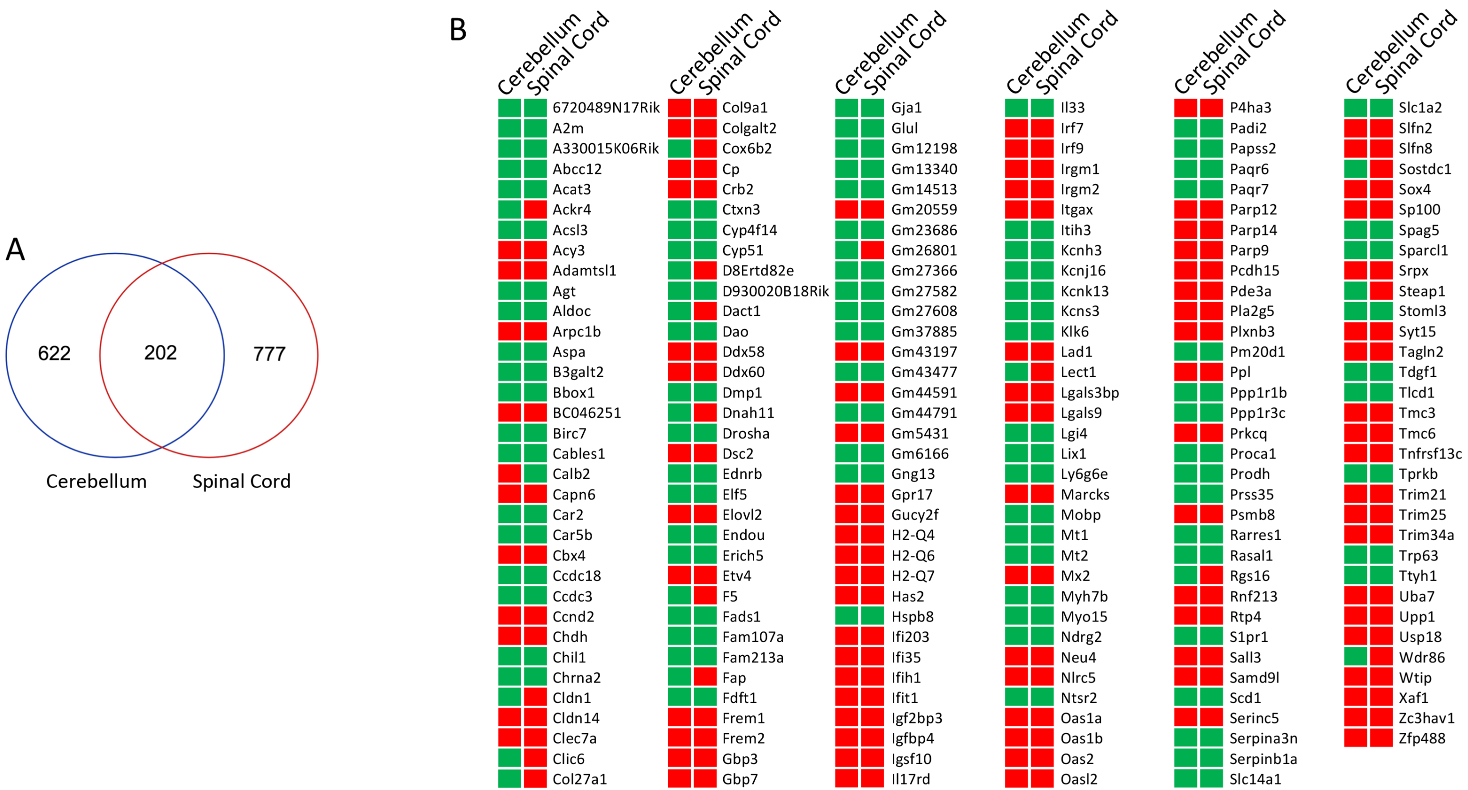

Supplementary Fig. 1. Graphic indication of the overlap of DEGs between CB and SC. Group 1 and group 2 pooled. A) Of 824 DEGs in CB and 979 in SC there were 202 that were shared. B) For the 202 shared genes, the indication is green if log2(FC) is negative, and red if positive. Genes listed in alphabetical order. All genes shown meet the criterion of AdjP<0.05, |log2(FC)|>0.585.
