# Supplementary material for: ALS-associated genes in SCA2 mouse spinal cord transcriptomes
Source: Hum Mol Genet. 2020 Apr 20;29(10):1658–72. doi: 10.1093/hmg/ddaa072 (PMC7322574; doi:10.1093/hmg/ddaa072)
Supplement: Supplementary_Figure_2_ddaa072 [file supplementary_figure_2_ddaa072.pdf]

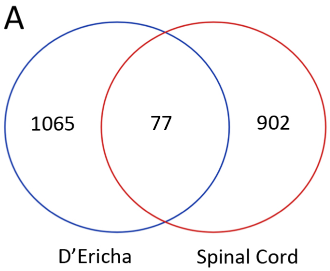

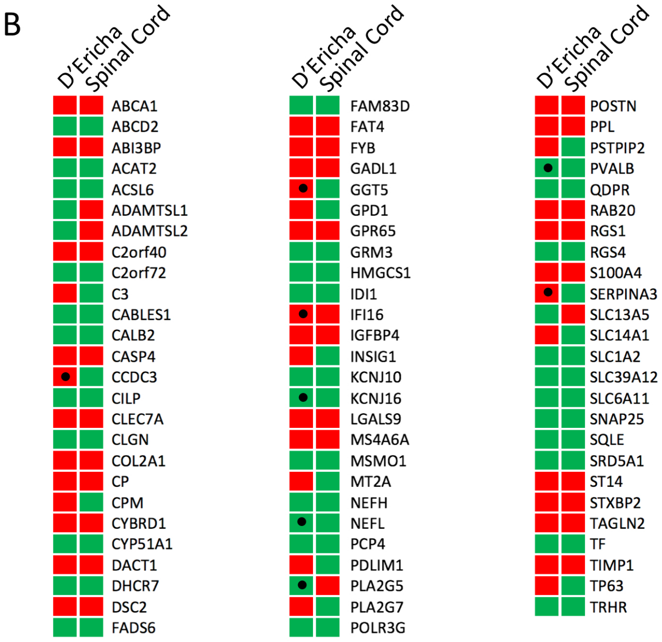

Supplementary Fig. 2. DEGs shared between D'Erchia et al. (2017) and SC pooled. A) Of 1142 DEGs in D'Erchia et al. (2017) and 979 in SC there were 77 that were shared. B) For the 77 shared genes, the indication is green if log2(FC) is negative, and red if positive. The cutoff criteria differed between the studies. While we used a cutoff of |log2(FC)|>0.585 and AdjP<0.05, the list in D'Erchia et al. (2017) that we collected this information from used AdjP<0.05 only. Employing the |log2(FC)|>0.585 criterion to the D'Erchia dataset would reduce the total DEGs by 113. Dots indicate D'Erchia DEGs with |Log2(FC)| values <0.585. Human gene names are shown.
