# Supplementary material for: ALS-associated genes in SCA2 mouse spinal cord transcriptomes
Source: Hum Mol Genet. 2020 Apr 20;29(10):1658–72. doi: 10.1093/hmg/ddaa072 (PMC7322574; doi:10.1093/hmg/ddaa072)
Supplement: Supplementary_Table_7_ddaa072 [file supplementary_table_7_ddaa072.pdf]

Supplementary Table 7. Gene sets referred to in Results and Discussion.

Annotation	Downregulated	Upregulated
**Spinal Cord, Untreated mice, BAC-Q72 vs Wildtype**		
Glial cell growth	*Agt, Aspa, Erbb3, Fa2h, Fgfr3, Gfap, Il33, Kcnj10, Lgi4, Metrn, Ndrg1, Plp1, Plpp3, Pou3f1, Qki, Tspan2*	*Dll1, Znf488*
Neuronal ensheathment	*Cldn11, Fa2h, Fgfr3, Kcnj10, Lgi4, Ndrg1, Mal, Ngfr, Plp1, Pou3f1, Qki, Tspan2, Ugt8*	
Fatty Acid Synthesis	*Agt, Acsm3, Elovl1, Fa2h, Fads2, Ggt5, Lpl, Pdk4, Plp1, Ptgis, Qki*	*Pla2g5*
Cholesterol metabolism	*Apoc1, Apoe, Lcat, Lpl, Ldlrap1, Lrp2, Pltp*	*Osbpl5*
Herpes simplex	*C3, Traf1*	*Ddx58, Eif2ak2, Hla-a, Iifh1, Irf7, Irf9, Oas1, Polr2a, Stat1, Tap1*
Interferon signaling		*Ifi35, Irf9, Oas1, Psmb8, Stat1, Tap1*
hepatic stellate cell activation	*A2m, Agt, Col6a5, Ednrb, Fgfr2, Lbp, Met, Ngfr*	*Col9a2, Col9a3, Col20a1, Myh3, Stat1*
LXR/RXR signaling	*Agt, C3, C4b, Il33, Lbp, Lcat, Lpl, Ngfr, Pltp*	
Th1/Th2 immune response signaling	*Fgfr2, Fgfr3, Icosl, Il33, S1pr1*	*Acvrlc, Dll1, H2-k1/HLA-A, Il12rb1, Il17rb, Stat1*
**Spinal Cord, Pooled group, saline treated, BAC-Q72 vs Wildtype, Yellow module**		
Potassium channels	*Kcna6, Kcnh3, Kcnj10, Kcnk13, Kcnn2, Kcns1, Kcns3, Kctd3*	*Lrrc26*
Glutamate receptor and glutamate synthesis genes	*Grin2c, Glul, Slc1a2, Grm3*	
Calcium channels	*Trpv3*	*Cacna2d4 (and Cacng4* and *Cacna1h were upregulated in the blue module)*
Chloride channels	*Ttyh1, Clic5*	*Clic6*
Mechanotransducer channels		*Tmc1, Tmc3, Tmc6*
Other ion transporters	*Slc4a4* (bicarbonate)	*Slc13a5* (citrate), *Atp7a* (copper)
Fatty acid synthesis	*Elovl1, Elovl5, Elovl7, Fa2h, Fads1, Fads2, Scd1*	*Acot1, Elovl2*
Hepatic stellate cell activation	*A2m, Agt, Ednrb, Fgfr2*	*Col9a1, Col9a2, Col9a3, Col20a1, Myh3*
Th2 pathway activation	*Acvr1c, Fgfr2, Fgfr3, Il33, Sqpr1*	*Dll1, Il12rb1, Prkcq*
**Spinal Cord, Pooled group, saline treated, BAC-Q72 vs Wildtype, Lightgreen module**		
Cholesterol synthesis	*Acat2, Cyp51, Dhcr7, Fdft1, Hmgcs1, Idi1, Insig1, Msmo1, Mvd, Nsdhl, Sc5d, Sqle*	
**Spinal Cord, Pooled group, saline treated, BAC-Q72 vs Wildtype, Midnightblue module**		
Innate immunity		*Apobec1, Ddx58, Ddx60, Gbp3, Herc6, Irf7, Irf9, Irgm1, Ifih1, Ifi35, Ifit1, Lgals3bp, Mx2, Oas1, Oas1a, Oas1b, Oasl2, Parp9, Parp 10, Parp12, Parp14, Nlrc5, Psmb8, Rtp4, Slfn2, Slfn8, Slfn9, Trim25, Trim34a, Zc3hav1*
**Spinal Cord, BAC-Q72, ASO7 vs Saline**		
Innate immunity and defense pathways		*C3, C4b, Clec7a, Ifi202b, Lgals3, Trem2, Tyrobp*
LXR/RXR and FXR/RXR activation		*C3, C4b, Il1rn*
Phagosome or lysosomal maturation		*Atp6v0d2, C3, Cd68, Ctss, Clec7a*
**Cerebellum, Pooled group, saline treated, BAC-Q72 vs Wildtype**		
Innate immunity		*Eif2ak2, Ddx58, Ifih1, Trim30a, Irf7, Irf9, Oas1a, Oas1b, Oas1c, Oas1g, Oasl1, Oas2, Oasl2*
**Ten spinal cord DEGs related to ALS progression identified by Recabarren-Leiva and Alarcón (2018)**		
AQP1, SLC14A1, MT1X, DSCR1L1, PCP4, UCHL1, GABRA1, EGR1, OLFM1, VSNL1		
**Nine SC DEGs in the Pooled group (saline treated BAC-Q72 vs WT) that were also found differentially expressed in SC of SOD1 mice by Bandyopadhyay *et al.*, 2013.**		
Hddc3, Cartprt, Nefl, Aldoc, Fth1, Mt1, Mt2, Pcp4, Gfap		
**Nine SC DEGs in the Pooled group (saline treated BAC-Q72 vs WT) that also appear in the ALSoD**		
Agt, Cst3, Dao, Fus, Grn, Nefh, Pcp4, Slc1a2, Znf512b		
