# Supplementary material for: ALS-associated genes in SCA2 mouse spinal cord transcriptomes
Source: Hum Mol Genet. 2020 Apr 20;29(10):1658–72. doi: 10.1093/hmg/ddaa072 (PMC7322574; doi:10.1093/hmg/ddaa072)
Supplement: Legends_to_Supplemtary_Tables_ddaa072 [file legends_to_supplemtary_tables_ddaa072.pdf]

Legends to Supplementary Tables

Supplementary Table 1. RNA-seq results comparing spinal cord extracts from BAC-Q72 mice (n=4) with non-transgenic littermate mice (n=4), age 19 wks. Mice in this experiment received no surgical treatments of any kind (designated “untreated group”) (see Table 1).

Supplementary Table 2. RNA-seq results comparing spinal cord extracts from BAC-Q72 mice (n=4) with non-transgenic littermate mice (n=4), sacrificed at age 19 wks (designated “early group”). Mice in this group received ICV injections of normal saline at 8 wks of age (see Table 1).

Supplementary Table 3. RNA-seq results comparing spinal cord extracts from BAC-Q72 mice (n=5) with non-transgenic littermate mice (n=3), sacrificed at age 34 wks (designated “late group”). Mice in this group received ICV injections of normal saline at 29 wks of age (see Table 1).

Supplementary Table 4. RNA-seq results comparing spinal cord extracts from BAC-Q72 mice (n=9) with non-transgenic littermate mice (n=7) of the pooled early and late groups (designated “pooled group”). Mice in this group were age 19-34 weeks at time of sacrifice and had received 5-10 wks treatments of normal saline (see Table 1).

Supplementary Table 5. WGCNA module membership for pooled group DEGs.

Supplementary Table 6. Pathway analyses for the different group comparisons and the significant WGCNA modules.

Supplementary Table 7. Lists of DEGs discussed in the text grouped by annotated pathways in which they function.

Supplementary Table 8. RNA-seq results comparing spinal cord extracts from BAC-Q72 mice (n=8) treated with ASO7, with BAC-Q72 mice (n=7) treated with normal saline (“TG ASO7 pooled group”). Mice in this group were age 19-34 weeks at time of sacrifice and had received 5-10 wks treatments of 175-210 µg ASO7 or normal saline (see Table 1).

Supplementary Table 9. RNA-seq results comparing spinal cord extracts from wildtype mice (n=4) treated with ASO7, with wildtype mice (n=9) treated with normal saline (“WT ASO7 pooled group”). Mice in this group were age 19-34 weeks at time of sacrifice and had received 5-10 wks treatments of 175-210 µg ASO7 or normal saline (see Table 1).

Supplementary Table 10. RNA-seq results comparing cerebellar extracts from BAC-Q72 mice (n=7) with non-transgenic littermate mice (n=9) of mice of the pooled early and late groups treated with normal saline (designated “CB pooled group”). On another tab of the Excel, RNA-seq results comparing cerebellar extracts from BAC-Q72 mice (n=8) treated with ASO7, with BAC-Q72 mice (n=7) treated with normal saline (“CB pooled ASO group”). Mice in these groups were age 19-34 weeks at time of sacrifice and had received 5-10 wks treatments of ASO7 or normal saline (see Table 1).

Supplementary Table 11. Intersection of DEGs in the CB pooled group vs cerebellar DEGs of 8 wk old mice presented in Dansithong et al., 2015 (ref. 14). GO and KEGG pathway analyses for the intersecting DEGs are also presented.

Supplementary Table 12. SCA2 mouse SC DEGs in the pooled dataset (Early + Late) that express proteins that are predicted by the Protein Atlas to be secreted, ranked by Log2FC.

Supplementary Table 13. List of primers and corresponding sequences.
